# Metabolite Profiling of Alzheimer's Disease Cerebrospinal Fluid

**DOI:** 10.1371/journal.pone.0031501

**Published:** 2012-02-16

**Authors:** Christian Czech, Peter Berndt, Kristina Busch, Oliver Schmitz, Jan Wiemer, Veronique Most, Harald Hampel, Jürgen Kastler, Hans Senn

**Affiliations:** 1 F. Hoffmann- La Roche, Pharmaceuticals Division, Basel, Switzerland; 2 Badisch Anylene Und Soda Fabriques (BASF SE), Limburgerhof, Germany; 3 Metanomics, Berlin, Germany; 4 Department of Psychiatry, Psychosomatic Medicine and Psychotherapy, Goethe University, Frankfurt, Germany; 5 Forenap Pharma, Rouffach, France; Case Western Reserve University, United States of America

## Abstract

Alzheimer's disease (AD) is a neurodegenerative disorder characterized by progressive loss of cognitive functions. Today the diagnosis of AD relies on clinical evaluations and is only late in the disease. Biomarkers for early detection of the underlying neuropathological changes are still lacking and the biochemical pathways leading to the disease are still not completely understood. The aim of this study was to identify the metabolic changes resulting from the disease phenotype by a thorough and systematic metabolite profiling approach. For this purpose CSF samples from 79 AD patients and 51 healthy controls were analyzed by gas and liquid chromatography-tandem mass spectrometry (GC-MS and LC-MS/MS) in conjunction with univariate and multivariate statistical analyses. In total 343 different analytes have been identified. Significant changes in the metabolite profile of AD patients compared to healthy controls have been identified. Increased cortisol levels seemed to be related to the progression of AD and have been detected in more severe forms of AD. Increased cysteine associated with decreased uridine was the best paired combination to identify light AD (MMSE>22) with specificity and sensitivity above 75%. In this group of patients, sensitivity and specificity above 80% were obtained for several combinations of three to five metabolites, including cortisol and various amino acids, in addition to cysteine and uridine.

## Introduction

Alzheimer's disease (AD) is a progressive and devastating neurodegenerative disorder of the brain characterized by loss of neurons and synapses, particularly in regions related to memory and cognition. AD is the most common form of dementia and its prevalence increases dramatically with age, from1% at the age of around 60 and up to 30% at the age of 85 or older [1].

Two main brain cortical lesions characterize AD: the accumulation of abnormally phosphorylated tau protein into paired helical filaments, known as neurofibrillary tangles (NFTs) within the neuronal cell and accumulation of beta amyloid outside the neurons in form of amyloid plaques and in the wall of cerebral blood vessels [2].

Diagnosis of AD relies on a combination of neuropsychological testing and the exclusion of other neurological, psychiatric or systemic diseases by the means of physical, neurological and laboratory examinations. The most often used neuropsychological criteria to diagnose AD were developed by the National Institute of Neurologic and Communicative Disorders and Stroke (NINCDS) and the Alzheimer's diseases and Related Disorders Association (ADRDA) workgroup in 1984 [3]. Because the clinical diagnosis of AD happens at a late stage of the disease, i.e. several years after the onset of the neuropathological alterations, there was an urgent need to revise these criteria in order to characterize the disease at a pre-dementia stage. It was proposed to take into account neuroimaging biomarkers and cerebrospinal fluid (CSF) analysis of amyloid beta or tau proteins in addition to the presence of deterioration in specific cognitive domains such as episodic memory [4]. In vivo imaging of amyloid deposits in the brain using specific PET ligands have made strong progress in the last few years and provide valuable tools for diagnosis, patient stratification and monitoring disease progression [5], [6]. Main drawbacks of PET are its high cost and restriction to highly specialized centers. Today, molecular CSF analyses appear as more promising, simpler and less expensive than imaging methods. The currently best CSF candidates are the amyloid-β (1–42) fragment and the Tau protein. Combinations of these markers reach sensitivity of about 90 to 95% and specificity about 85% (for review see [7]). However, using these CSF markers there is still huge overlap with other forms of dementia. and the capacity of these parameters to identify the therapeutic efficacy of new disease-modifying treatments has not yet been proven [8]. Taking into account the multifactorial nature of AD, it is likely that the same clinical manifestations are underlain by different neuropathological mechanisms. Thus, combination of several biological markers acting at different physiological levels can bring complementary information for diagnostics of various disease phenotypes and for monitoring biological drug effects.

High-density “Metabonomics/Metabolomics” approach offers the prospect of efficiently distinguishing individuals with particular disease or toxic states on the basis of their metabolite profile in biofluids. Emerging specific analytical technologies, including nuclear magnetic resonance (NMR) and liquid chromatography-tandem mass spectrometry (LC-MS) are particularly relevant to produce unbiased metabolic signatures of biofluids and tissues (for a review see [9]). In particular for CSF the situation is especially favorable since many endogenous metabolites in this compartment are known and have spectroscopically been assigned [10].

Here we show the metabolic profile in human CSF samples of AD patients and age-matched healthy controls. This approach attempts to identify candidates for biomarkers traced to particular metabolites or pathways specific for AD or the underlying neurodegenerative process and is used as a starting point for further validation in independent sample sets.

## Materials and Methods

### Participants and ethics

Subjects of both sexes aged of 40 years or above were recruited in five different clinical centres in Europe: One in Germany, one in France, one in Switzerland, and two in Sweden, after approval of the protocol by the ethics committee of the corresponding countries: University Clinic Munich, CCPPRB Alsace I Strasbourg, Ethikommission beider Basel, University Clinic Huddinge and Uppsala. All procedures were conducted according to the principles expressed in the declaration of Helsinki and all subjects gave their written informed consent. In case patients were considered not to have the capacity, inform consent was given by relatives.

Diagnosis of AD was made according to the NINCDS-ADRDA and DSM IV criteria. The Hachinski ischemia scale was used to exclude dementia of the vascular type. Controls were age-matched cognitively normal persons. For all subjects, the following drugs were prohibited: anticoagulants or anti-inflammatory (COX-2 inhibitors) for at least 3 months, symptomatic treatment for cognitive disorders within 30 days, treatments for depression, schizophrenia or anxiety if not stabilized for at least 30 days. Details on demographic and clinical characteristics are given in Table 1.

**Table 1:** Characteristic of study and participants for all centers and core centers.

CHARACTERISTIC	Light to mild AD (MMSE>22)	Moderate to Strong AD (MMSE 14–22)	Control
No of participant^1^ (all)^2^	53	26	51
Participant (core)^3^	47	24	30
Gender M/F (all)	23/30	12/14	24/27
Gender M/F (core)	21/26	11/13	13/17
Age in years (all)	69.7 (±9.5)	69.6 (±10.1)	63.1 (±7.7)
Age in years (core)	68.5 (±9.3)	68.2 (±9.3)	65.1 (±8.8)
MMSE (all)	25.8 (±1.7)	19.7 (±2.6)	na
MMSE (core)	25.8 (±1.6)	19.7 (±2.6)	na

1 sample volume: 500 µl CSF; for 4 patients with MMSE>22 <500 µl were available leading to incomplete data sets.

2 “all” means from five different clinical centers in Europe, two of them providing only samples from controls (21) and AD (8), resp.

3 “core” includes only three from five centers which provided both control and AD samples.

### Study procedures

A MRI examination or PET scan was performed in patients if these examinations were not realized within the last 12 months. CSF samples were taken according to standard procedures in both patients and healthy subjects for metabolite profiling and determination of the concentrations of Abeta42 and tau protein. Samples were collected using the same type of tubes, labeled by Roche and shipped to the different clinical centers.

#### Metabolite Profiling

Three types of mass spectrometry analysis were applied to all samples: GC-MS (gas chromatography-mass spectrometry) and LC-MS/MS (liquid chromatography-MS/MS) were used for broad profiling, as described elsewhere [11]. SPE-LC-MS/MS (solid phase extraction-LC-MS/MS) was applied for the determination of catecholamine and steroid levels. Proteins were removed from CSF samples by precipitation. Subsequently polar and non-polar fractions were separated for both GC-MS and LC-MS/MS analysis by adding water and a mixture of ethanol and dichloromethane. For GC-MS analysis, the non-polar fraction was treated with methanol under acidic conditions to yield the fatty acid methyl esters derived from both free fatty acids and hydrolyzed complex lipids. The non-polar and polar fractions were further derivatized with O-methyl-hydroxyamine hydrochloride and pyridine to convert oxo-groups to O-methyl-oximes and subsequently with a silylating agent before analysis [12]. For LC-MS analysis, both fractions were reconstituted in appropriate solvent mixtures. HPLC was performed by gradient elution using methanol/water/formic acid on reversed phase separation columns. Mass spectrometric detection technology was applied which allows target and high sensitivity MRM (multiple reaction monitoring) profiling in parallel to a full screen analysis (Patent: WO2003073464). For GC-MS and LC-MS/MS profiling, data were normalized to the median of reference samples which were derived from a pool formed from aliquots of all samples to account for inter- and intra-instrumental variation. Steroids, catecholamines and their metabolites were measured by online SPE-LC-MS/MS [13]. Absolute quantification was performed by means of stable isotope-labelled standards.

### Statistical analysis

Simple (t-test) and multi-factor ANOVA models capturing the factors gender and age in addition to disease status were estimated for log10-transformed relative concentrations (Ratios) of all semi-quantitative SQ-metabolites (see “Types, number and quality of analytes measured”) and absolute CSF concentrations of (p-)Tau and amyloid beta proteins (Analysis conducted by the statistics software R) [14]. ANOVA models were applied to correct metabolic data for age and gender and to select metabolites with high diagnostic potential. The resulting gender- and age-corrected metabolites were combined (pairs to quintets) and the combinations examined by binary disease status classification using penalised logistic regression (PLR) [15] and receiver operating characteristics (ROC) analysis [16]. Metabolite combinations were evaluated by area under curve (AUC) values of their corresponding PLR classifier. AUC values indicate the extent to which samples can be correctly classified without specifying a classification threshold.

Multivariate statistics is a form of statistics encompassing the simultaneous observation and analysis of more than one statistical variable. Multivariate statistics was performed by using Simca P+ software (Umetrics, Umea, Sweden). The data analysis included PCA (principal component analysis) and OPLS-DA (orthogonal projections to latent structures-discriminant analysis). Prior to PCA and OPLS-DA, data were scaled to unit variance introducing a common scale for all metabolites independent of their absolute amount of variance. Thereby, the resulting models obtained robustness, i.e. they could not be dominated by a single or few high-variance metabolites. PCA is an explorative unsupervised multivariate analysis method used for the detection of trends, patterns and groupings among samples and variables. In addition, it is also useful for the detection of biological outliers ( = deviating samples that are extremely different from the rest of the data set). Therefore, PCA was used both at the level of quality control as well as to obtain an unbiased overview of the metabolite profiling dataset. In order to focus on differences between specific sample groups, i.e. AD patients and healthy controls, OPLS-DA as a supervised multivariate approach was used for prediction and classification purposes. Hereby, specific prior knowledge is incorporated by assigning samples to the respective classes. The principle of OPLS-DA is to well approximate metabolic data and class assignments, and at the same time to maximize their correlation. Abeta 42 and tau/pTau were measured using commercially available ELISA assays (Innogenetics, Belgium).

## Results

### Study and sample details

Two of the participating centers provided only samples from healthy controls (n = 21) and from AD patients (n = 8), respectively. The samples were collected at various time points between 2000 and 2005. Disease samples originated from AD patients with varying degree of dementia as indicated by different scores from the MMSE: light or mild AD (MMSE>22), or strong AD (MMSE 14–22). In total 79 CSF samples from AD patients and 51 CSF samples from healthy subjects were analyzed. In addition the concentration of the AD protein biomarkers amyloid beta 42, p-Tau 181, and Tau were known for a subset of 43 samples, 19 of which were from the control group, 17 belonged to the group of light AD patients and 7 to the group of strong AD patients. The distribution of the samples over the various patient groups is summarized in Table 1.

### Types, number and quality of analytes measured

**Figure 1:**
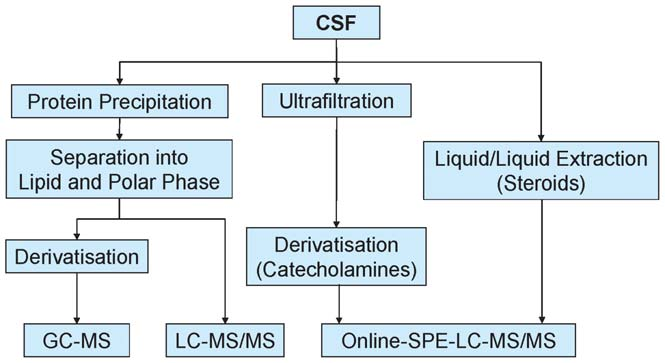
The metabolite profiling techniques applied to the analysis of CSF samples.

An overview of the various analytical methods that were applied for metabolite profiling of the CSF samples is schematically shown in Figure 1. In order to gain maximum information, the polar phase was subjected to different liquid chromatographic separation conditions as well as different ionization techniques for mass spectrometric detection. In LC-MS/MS, a targeted profiling technology was applied which allowed for high sensitivity Multiple Reaction Monitoring (MRM) in parallel to untargeted full screen profile analysis [11]. For the lipid fraction, tandem-MS experiments made the observation of specific transitions possible that are typical for certain lipid classes, especially phospholipids.

A list of all structurally assigned metabolites with their relative change in the different groups is provided in Table S1.

In addition, a number of catecholamines and steroids that are discussed in the context of AD and are present in CSF at very low concentration levels (ng/ml) were analyzed (Table S2). These analytes are hardly accessible with standard metabolite profiling techniques. In order to get access, two on-line SPE-LC-MS/MS were applied that allow for absolute quantification by use of calibration standards. The experimental details of all the separation and metabolite profiling techniques have been documented in the previous section.

Statistical analysis is confined to 343 analytes which were measured at concentrations being well above the limit of quantification (LOQ) allowing SQ analysis. This is to make sure that results based on SQ-analyte data are analytically reliable, i.e. SQ-analytes are used to detect quantifiable differentiating features between sample collectives. The group of SQ analytes comprised 80 known metabolites, i.e. structurally assigned (target) metabolites, including 15 hormones/neurotransmitters (steroids, catecholamines and related) for which absolute quantification was provided, and 260 known-unknowns. The latter are those found in each sample with a defined retention time and mass spectrum but the identification via chromatopraphic comparison or isolation and structural elucidation has not yet been accomplished. Typically the number of known-unknowns is very much higher in biological samples than the knowns.

### Univariate statistical evaluation

Univariate statistical t-test analysis of the SQ-analytes was carried out for females and males. The results are shown in table 2 for two different significance thresholds depending on gender and disease status: light AD with MMSE>22, strong AD with MMSE 14–22 and all AD patients. Detailed statistical analysis data for all structurally assigned metabolites is shown in Table S1.

**Table 2:** Number of semi-quantitative analytes significantly altered in patients diagnosed with Alzheimer's disease obtained from t-test analysis in dependency of gender and significance threshold p (in grey: number of significant changes equal or below expected false-positive rate).

significance threshold p	Females	AD MMSE>22 vs. controls	AD MMSE 14–22 vs. controls	AD all vs. controls
<0.05	All Centers	**33**	**23**	**29**
	Core Centers only	**20**	*15*	*17*
<0.01	All Centers	**9**	**4**	**13**
	Core Centers only	*3*	*0*	*3*

significance threshold p	Males	AD_MMSE>22_vs. controls	AD_MMSE 14–22_vs. controls	AD_all_vs. controls
<0.05	All Centers	**24**	**21**	**27**
	Core Centers only	*13*	**19**	*13*
<0.01	All Centers	**4**	**6**	**11**
	Core Centers only	*1*	**6**	*4*

The univariate statistical analysis revealed that female AD patients show a higher number of significant metabolic changes than male AD patients compared to healthy controls. This observation is in line with larger sample groups yielding higher test power and larger age-imbalance for females than for males. Furthermore, females diagnosed with light AD (MMSE>22) exhibit more alterations than those diagnosed with advanced AD (MMSE 14–22).

**Figure 2:**
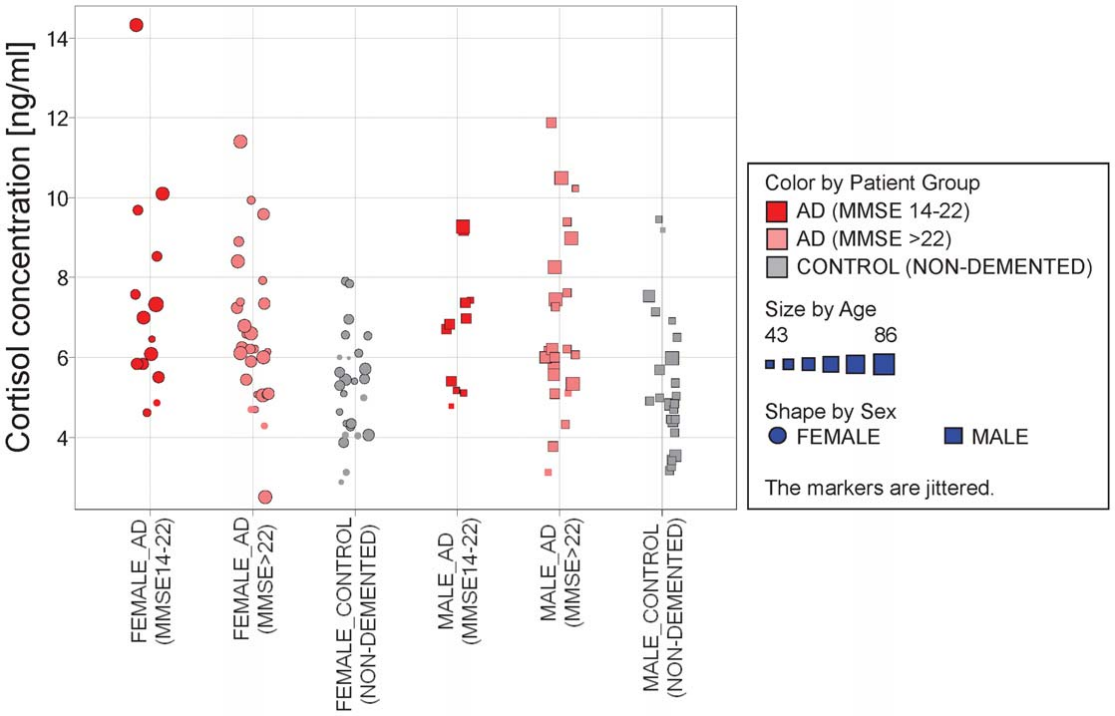
Increase of cortisol concentration observed for AD patients compared to healthy controls.

**Figure 3:**
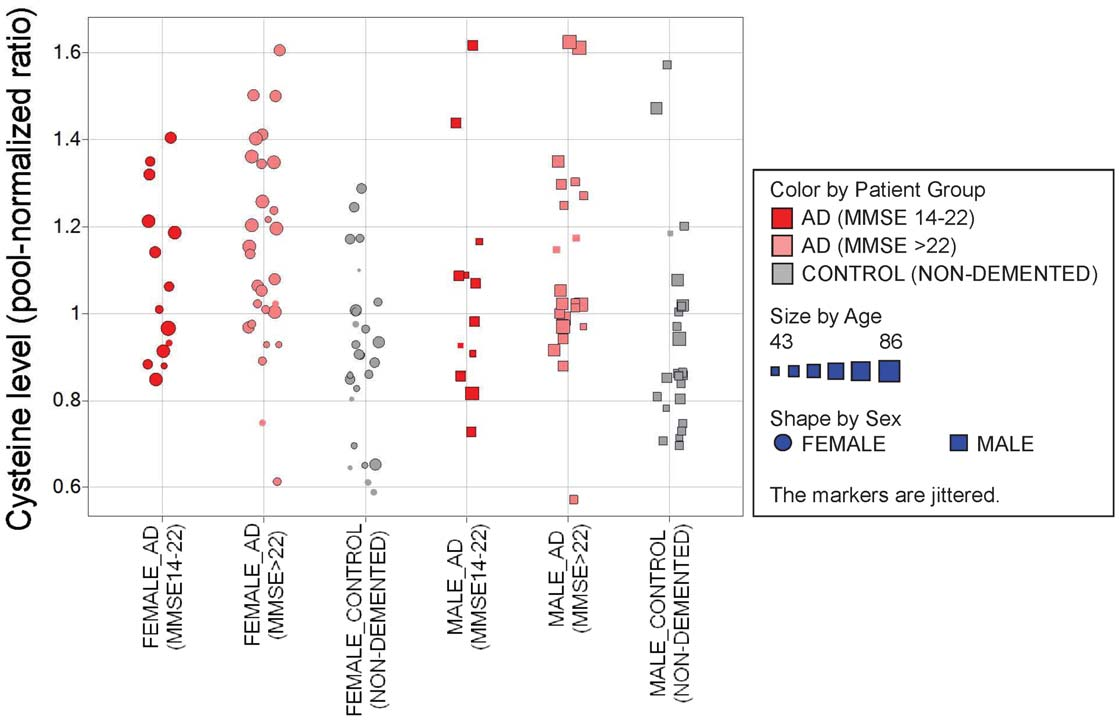
Increase of relative cysteine levels observed for AD patients compared to healthy controls: most prominent changes detected in AD patients with MMSE 22.

The intersection of significantly changed analytes (p<0.05; AD_all_ vs. controls) for females (29) and males (27) comprises 8 metabolites of which 7 are knowns, namely cysteine, uridine and five hormones/neurotransmitters: cortisol, 3-methoxy-4-hydroxy phenylglycol (MHPG), dopamine, noradrenaline and normetanephrine. In general, the AD related metabolic changes are light to moderate, e.g. increase by up to ∼40% in the case of cortisol (figure 2) or increase by 20–25% primarily in light AD patients for cysteine (figure 3). Further examples are uridine and MHPG which both are slightly decreased by ∼10–20% in AD patients compared to healthy subjects. In the case of cortisol the degree of change seems to be related to the severity of AD, i.e. alterations compared to controls are higher in the group of AD patients exhibiting an MMSE score of 14–22 than in patients with MMSE>22 (figure 2).

**Figure 4:**
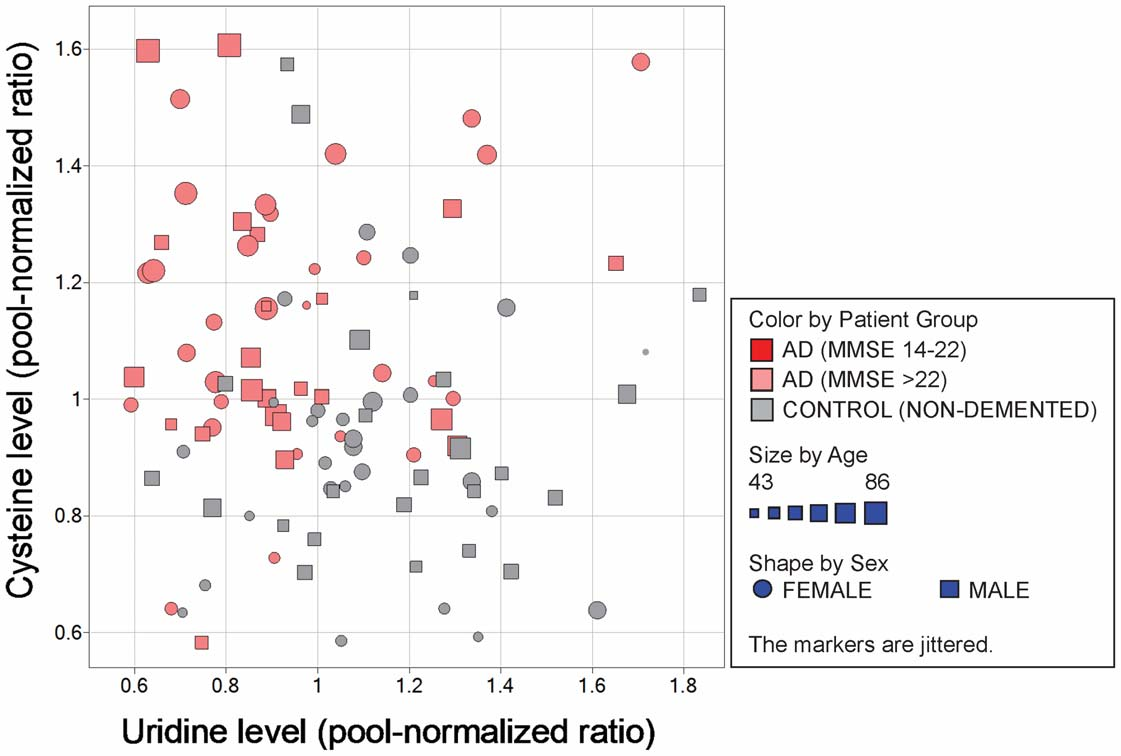
CSF cysteine and uridine levels of patients (females and males) exhibiting light AD (MMSE>22) compared to healthy controls: ∼75% of all samples can be correctly assigned to the respective patient group by using relative ratios for both metabolites.

Most metabolite changes were particularly observed in the light AD groups (table 2). Interestingly, the combination of cysteine elevation and uridine decrease is best suited for distinguishing these AD patients from healthy controls (figure 4).

### Multivariate statistical analysis of SQ-analytes

Principal component analysis (PCA) for all 130 samples based on the 343 SQ-analytes showed partial separation of gender in the score plot, mostly along principal component 2 (data not shown). Further, samples from males and females exhibit an influence of age and/or disease on the metabolite profile. In the PCA score plot AD patients, especially the elder ones, are shifted approximately orthogonal to the gender related segregation, i.e. primarily along principal component 1.

**Figure 5:**
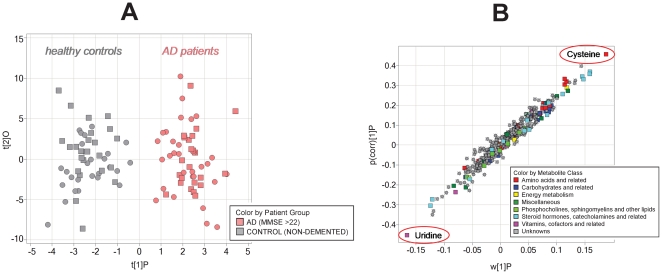
Determination of analytes contributing significantly to separation between AD patients and healthy controls by O-PLS-DA of the CSF data set. (A) OPLS-DA score plot for light AD patients (MMSE>22) vs. controls. (B) OPLS-DA coefficient plot for light AD patients (MMSE>22) vs. controls. Cysteine and uridine are the most relevant analytes for separating light AD patients (MMSE>22) from healthy subjects.

Analytes contributing significantly to separation between AD patients and healthy controls were determined by O-PLS-DA of the CSF data set (see material and methods). The resulting score plot, involving all 343 SQ-analytes in the calculation, is shown in figure 5a where separation between AD patients (MMSE>22) and healthy controls was observed (cross-validation performed; Q^2^ = 0.23).

The relative contribution to class separation is visualized for the 343 analytes in the corresponding O-PLS coefficient plot (figure 5b). The prominent role of cysteine and uridine for discriminating both groups, a result which was already deduced from univariate statistics, is confirmed. A similar comparison of the SQ-analyte profile of patients exhibiting strong AD with those of healthy subjects by O-PLS-DA leads to an equally good class differentiation in the score plot (data not shown). Again cortisol was found to be the most prominent analyte contributing to separation in addition to dopamine, sorbitol and several unknowns.

### Linear modeling of systematic differences in age, gender and disease status

**Figure 6:**
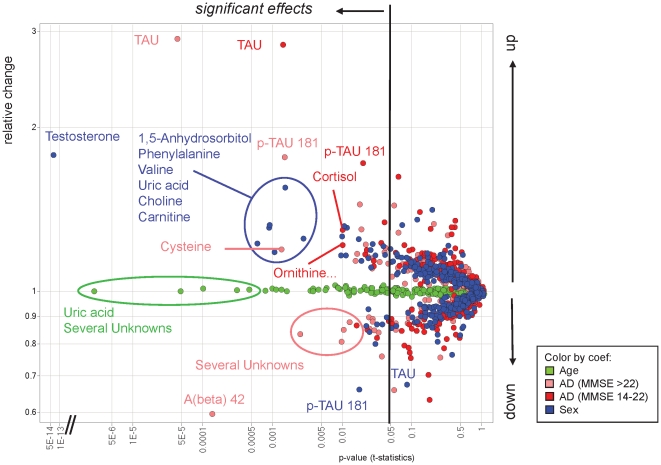
Significance levels and relative changes with respect to influencing factors gender, age and disease status (light AD; strong AD) are shown. All SQ 343 analytes measured by metabolite profiling, and the proteins TAU, p-TAU 181 and amyloid beta 42 were included in the linear modeling calculation. In the case of gender-related correlations (blue circles) relative changes >1 indicate increased levels in males compared to females (logarithmic scale for both axes; relative change for age-related effects are not-to-scale).

Systematic differences such as age, gender and center between AD patients and healthy controls are inherently present in this data set (table 1),Therefore, systematic age- and gender-related effects were corrected by applying linear modeling employing the relative concentrations (ratios) of all 343 SQ-analytes (see material and methods for details). In addition to metabolite profiling data, total Tau, p-Tau, and amyloid beta 42 concentrations were included as far as available; however center correction was not reasonable in view of the limited availability of protein data in this study. The result of the calculation for all centers with respect to the influencing factors age, gender and disease status (light; strong AD) is given in figure 6. The relative changes and significance level for the 343 SQ metabolites and the proteins Tau, p-Tau 181 and amyloid beta 42 are shown. The most significant metabolic effects are related to sex (blue) and age (green): increased levels of testosterone and, to a lesser extent, elevated levels of 1,5-anhydrosorbitol, phenylalanine, valine, choline, carnitine and uric acids in males, and increased levels of uric acid and several unknowns with respect to age. The most significant disease related effects pertain to the proteins Tau/p-Tau (increase) and amyloid beta42 (decrease) as well as several analytes identified by metabolite profiling, particularly cysteine, several unknowns, cortisol and ornithine, including minor components arginine and citrulline, most of which are best correlated with light AD.

Almost 50% of healthy control samples from males originated only from one center which did not contribute any AD sample to the study. On the other hand there was also one center providing only AD samples and no controls. To account for center effects due to providing either no control or no AD samples a similar calculation was done employing only the data from the three core centers which provided samples from both, healthy controls and AD patients (result not shown). This calculation included only metabolite profiling data and not protein data because protein data was insufficient for this sample subset (5 female and 2 male values). The most prominent disease effects on metabolites were identified for cysteine (increase), taurine (increase) and for uridine, mannose and several unknowns which all decreased.

### Potential markers from metabolite profiling by discriminant analysis

In order to identify the most promising AD-related parameters corrected for age- and gender-related effects according to their univariate performance in linear modeling, the significance levels for all center calculation (fig. 6) were related to those obtained for core center results, both for correlation with light AD status and strong AD status, respectively. Then, the set of analytes exhibiting sufficient univariate performance, i.e. having p values <0.1 in the ‘all’ center and <0.2 in the ‘core’ center models, was selected. In case of light AD the metabolites fulfilling these criteria were 5 unknown and 11 known analytes, namely cysteine, tyrosine, phenylalanine, methionine serine, pyruvate, taurine, creatinine, cortisol, dopamine and uridine. Much less analytes showed comparably strong correlations with later stages of AD (MMSE 14–22), namely 9 unknowns with two of them also appearing in the light AD model above, and two knowns, ornithine and cortisol.

The subsequent discriminant analyses focused on light AD (MMSE>22), i.e. on the 16 analytes selected after correction of age- and gender-related effects (see above). The rationale for this lies in the limited number of samples from patients exhibiting strong AD (MMSE 14–22) and the substantial interest in diagnosing AD particularly at early stages, combined with the fact that metabolite alterations were more prominent in the group of light AD patients. The most powerful metabolite pairs, triplets, quartets and quintets from the 16 identified metabolites were evaluated from all possible metabolite combinations by applying multivariate classification (PLR). The top five metabolite combinations with highest ROC AUC values are depicted in table 3 for each number of metabolites. The ROC AUC values are biased upwards by the selection of best metabolite combinations from the large set of all metabolite combinations of given dimension. Unbiased ROC AUC performance estimates for the biomarker nominations of Table 3 require ROC analysis of new samples from an additional validation study.

**Table 3:** Metabolite combinations with highest predictive potentials according to ROC AUC values starting with most promising metabolite combination (“rank”) for each combination type (pairs, triplets, quartets, quintets). Multivariate classification was based on PLR with combinations of the 16 metabolites selected from and gender- and age-corrected by univariate statistical ANOVA modeling.

combinations	rank	Uridine	Cortisol	Cysteine	Dopamine	Methionine	Phenylalanine	Serine	149401061.Unknown	149401096.Unknown	39400528.Unknown	AUC
pairs	1	x		x								0.793
	2	x	x									0.770
	3		x	x								0.770
	4	x							x			0.763
	5		x								x	0.763
triplets	1	x	x	x								0.825
	2	x		x					x			0.816
	3	x	x								x	0.813
	4	x		x	x							0.806
	5	x		x				X				0.805
quartets	1	x	x	x						x		0.836
	2	x	x	x			x					0.833
	3	x		x	x				x			0.832
	4	x		x					x	x		0.831
	5	x	x	x				X				0.831
quintets	1	x	x	x			x			x		0.845
	2	x		x	x				x	x		0.843
	3	x	x	x				x		x		0.841
	4	x	x	x		x				x		0.840
	5	x	x	x						x	x	0.839

**Figure 7:**
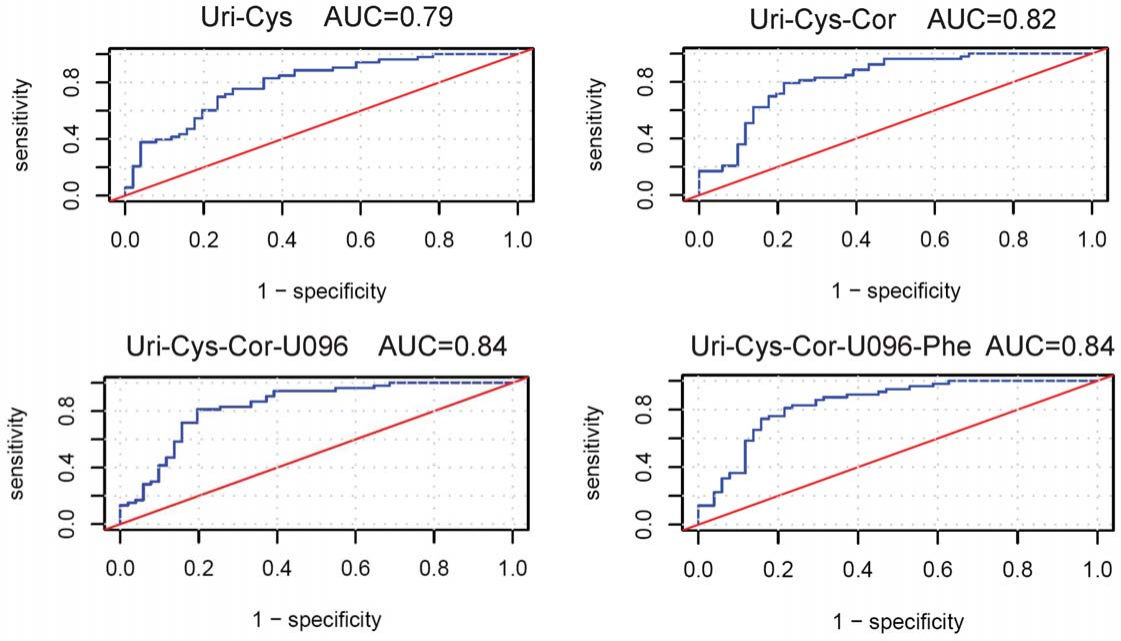
Receiver operating characteristics (ROC) curves with AUC values for best analyte pair (top left), triplet (top right), quartet (bottom left) and quintet (bottom right). Sensitivity (true positive rate) and 1 – specificity (false positive rate) relate to correct prediction of AD (MMSE>22).

The best metabolite pair (cysteine+uridine) allows AD prediction with e.g. ∼75% sensitivity and ∼75% specificity. In general, >80% sensitivity with >80% specificity is observed for quite a few metabolite combinations, mainly quartets and quintets, e.g. the quartet uridine, cortisol, cysteine and unknown U096 (figure 7). In comparison, AUC values between 0.85 to 0.97 are obtained for single to combinations of all three proteins Tau, P-T and amyloid beta42. However, the predictive potential might be overestimated in the case of proteins, as no center corrections could be applied due to the limited number of cases. That center dependence seems to exist was concluded from a PCA on total Tau, pTau and amyloid beta42 for 17 patients with light AD and 19 controls, where 12 of the controls came from the one center which provided only control samples. Controls originating from this center appeared to be more different compared to AD samples than observed for the controls from the other centers.

Aside from the combinations shown in Table 3 which are dominated by cysteine, uridine and cortisol, further combinations with slightly lower AUC values exist and which are thus less dependent on the performance of these three metabolites, e.g. the quartets cortisol, serine, unknown U528 and either unknown U096 or dopamine with AUC values 0.806 and 0.804, respectively. The amino acid focused quintet cysteine, serine, phenylalanine and tyrosine together with uridine has an AUC value of 0.799.

## Discussion

Alzheimer's disease (AD) is characterized by progressive neurodegeneration – large scale atrophy of brain tissue in many brain regions. It is evident that different brain structures are affected differentially during progression starting with hypothalamic structures before progressing to the cortex. It can be assumed that these changes in neuronal integrity as well as the underlying pathology lead to changes in brain metabolism which is reflected in the composition of metabolites in CSF.

In the current study we applied very sensitive and quantitative analytical tools to detect these changes in brain metabolism. We show that using metabolite profiling; statistically significant differences in CSF between AD patients and controls can be detected. In general these differences are moderate, the increase is only up to around 40% (for cortisol, see below) and in addition there is a considerable overlap between the sample groups. However, the data strongly suggest that chronic neuronal changes in the brain of AD patients result in changes in brain metabolism which can be detected in CSF.

It is particular interesting to look into neurotransmitter systems, since these directly reflect activity and integrity of neuronal networks.

Acetylcholinesterase inhibitors are currently used in AD therapy to address cognitive symptoms. As early as in the mid-70s, it had been established that choline acetyltransferase activity is much more reduced in brain tissue from AD patients than in age-matched controls – an observation commonly known as cholinergic deficit in AD [17]. Due to its rather low concentration, a direct confirmation of resulting lowering of acetylcholine in CSF could be demonstrated only in the mid-90s [18]. Observed acetylcholine concentrations in normal patients reach 30±10 nM, compared to 8±4 nM in AD patients. Acetylcholine is a highly charged small molecule that was could not be included in our metabolite panel, therefore we have no estimate of this difficult to analyze established marker.

However, while cortical cholinergic neurons suffer most pronounced losses, other types of neurons are progressively affected as well and this is reflected in changes in the concentration of norepinephrine (NE) and its major metabolite 3-methoxy-4-hydroxy phenylglycol (MHPG) as we have observed in our study. It has been established that up to 80% of the neurons located in the locus coeruleus are lost in AD [19]. As a result, the concentration of norepinephrine (NE) at the sites of locus coeruleus neuronal projections in the frontal and temporal cortex is substantially lowered [20]. It appears that increased NE concentrations in CSF as we have observed in our study are a reflection of compensatory effects, connected with higher activities per neuron and higher secretion into the CSF. Paradoxically, the majority of studies find different results in CSF – ranging from unchanged levels to a marked increase of NE or its major metabolite 3-methoxy-4-hydroxy phenylglycol (MHPG). Loss of neurons in the locus coeruleus occurs progressively with increasing age, but older subjects have commonly higher levels of NE in CSF than young adults. However, NE concentrations in CSF of AD patients can be much higher than those seen in cognitive normal elderly patients. It appears that noradrenergic neurons can re-innervate de-innervated cholinergic brain regions [21]. Moreover, NE concentrations in CSF due to apparent NE- dependent compensation can be induced by factors such as insulin and can lead to improved mental control and memory recall [22].

Finally, in normal aging, tyrosine hydroxylase and aromatic acid decarboxylase enzyme activities decrease but monoamine oxidase activity increases. Therefore, dopamine and its metabolic product HVA are expected to be lowered in CSF of the elderly, and AD disease progression should add to the deficit. Indeed, AD patients have slightly (18–27%) lowered dopamine levels in cortical tissues and the hippocampus [20], [23]. Compensation mechanisms can increase the activity of the remaining neurons to an extent that the levels of dopamine or HVA in CSF appear unchanged until late into the course of the disease [24]. However, slightly lowered levels are observed with appropriate selection of the patient population [25]. Our study identifies dopamine as one of the few catecholamines differentiating AD from controls, which is consistent with the described observations in literature.

Hyperactivity of the HPA axis and increased cortisol levels in CSF and serum of AD patients has been described previously in AD patients in several studies [26], [27], [28]. Here we confirm these earlier finding. Interestingly, there is no significant difference between mean cortisol levels in the light dementia group compared to the more severe demented patients. This argues that cortisol may reflect early pathological changes in the brain. It has been shown that cortisol undergoes circadian rhythm and could be also induced by the stress of the lumbar puncture itself [29]. In our study there was no systematic bias in the time point of CSF sampling between AD patients and controls. However it could well be that AD patients are more responsive to stress than healthy control and this again would argue for deregulation of the HPA axis.

Uridine is a nucleoside and as such part of RNA. In addition, it is besides choline and DHA also one of the precursor of phosphatidylcholine (PC) which is synthesized via the Kennedy cycle. PC is the major component of cellular membranes. It has been reported that dietary supplementation of these precursors increase the synthesis of phosphatides in the brain, the number of synapse and also promotes the formation of dendritic spines in the hippocampus (for review see [30]). The observed reduction of uridine in the CSF of AD patients could reflect the reduced synaptic plasticity and neuronal deficits.

There have been many reports that metabolic dysfunction is linked to the pathology AD. The incidence of diabetes is increased in AD and it has been hypothesized that Insulin resistance in the brain contributes to AD pathology (for review see [31]). Interestingly in our data set we do not see any significant differences in glucose in CSF of AD patients or metabolites linked to insulin resistance. It might well be that these proposed metabolic abnormalities are not reflected in CSF.

Homocysteine as well as cysteine have been reported to be elevated in plasma and serum of AD patients [32]. Many studies have shown that increased homocysteine is associated with an increased risk of cognitive impairment and dementia, however this is still discussed controversially (for review see [33]). Homocysteine levels are not changed in the CSF of AD patients [34]. However, in the present study we detect a significant increase of cysteine in CSF of AD patients. Homocysteine can be metabolized to cysteine and the observed increased levels of cysteine could reflect a misbalance in the homocysteine metabolic system. Further studies in possibly larger patient cohorts are needed to confirm these findings. One clinical study has shown that lowering of homocysteine in plasma by Vitamin B12 slows the rate of brain atrophy, however without effect on cognitive parameters [35]. These data argue again that the increase in cysteine levels in AD patients as observed in our study is a marker of neurodegeneration in the brain of AD patients.

Our study complements efforts to detect changes in CSF protein composition caused by the neurodegenerative alterations. Indeed, there have been numerous studies (reviewed in [36] [37] using novel proteomics approaches that include not only unbiased analysis of CSF using protein separation techniques and mass spectroscopy, but also targeted approaches with multiplex panels of specific analytes. While these studies have found a number of candidate combinations that could improve on the “core” protein biomarkers phospho-tau and amyloid beta 42, none has been validated enough to be included in clinical practice. Many of the new protein biomarker candidates seem to reflect late degenerative mechanisms (gliosis or inflammation).

When compared to the published proteomics literature, our metabolomics approach has a number of distinct advantages: protein content of the CSF is highly variable and dominated by a number of abundant plasma proteins that mask a small contribution of neurons, the number of proteins to be measured (>5000 species) is much larger than the potential number of metabolites (a few hundreds), which has implications on the dimensionality of the experiment and the multiple testing corrections that need to be applied, and finally, the exchange of many small molecules between CSF and other body fluids is restricted and reflects the unique metabolic situation in the brain.

It should be noted, that the data we present here are by no means biomarkers to be used in clinical practice. In particular, since the marker performance even of the best metabolite combinations is not exceeding the performance of the known protein markers Abeta 42 and Tau in CSF. Furthermore, statistical tools (metabolite selection, classification, ROC analysis) were applied to find metabolite combinations with highest diagnostic performance on the current datasets. The ROC AUC values should not be regarded directly as performance estimates because of a selection bias that can be quite strong especially for -omics datasets with many features (“trap of overfitting”). A natural next analysis step would be to test the listed metabolite combinations on new data to get real estimates of their performance. In any case, the data set has shown that metabolite profiling technology can be applied to analyze the signature of brain metabolism in humans during chronic disease and that changes can be detected reflecting chronic disease processes in the brain such as Alzheimer's disease pathology.

## Supporting Information

Table S1 **Table of all structurally assigned metabolites with their relative change in the different groups shown as Fold difference between the groups and the results of the statistical analysis of each data set showing the p-value.**
(PDF)

Table S2 **The table S2 shows the results as mean of the group values of the metabolites measured in CSF with absolute quantification. All values are in ng/ml.**
(PDF)
