# Supplementary material for: Metabolite Profiling of Alzheimer's Disease Cerebrospinal Fluid
Source: PLoS One. 2012 Feb 16;7(2):e31501. doi: 10.1371/journal.pone.0031501 (PMC3281064; doi:10.1371/journal.pone.0031501)
Supplement: Table S1 — Table of all structurally assigned metabolites with their relative change in the different groups shown as Fold difference between the groups and the results of the statistical analysis of each data set showing the p-value. (PDF) [file pone.0031501.s001.pdf]

Supplement Table 1

Quantification method	Metabolite Name	Metabolite Class	Global perspective (all centers)																	
			Fold difference (AD vs. Controls)									t-test (p-value) based on log-transformed data								
			Female + Male			Female			Male			Female + Male			Female			Male		
			AD patients (all) compared to controls	AD patients (MMSE > 22) compared to controls	AD patients (MMSE 14-22) compared to controls	Female AD patients compared to controls	Female AD patients (MMSE > 22) compared to controls	Female AD patients (MMSE 14-22) compared to controls	Male AD patients compared to controls	Male AD patients (MMSE > 22) compared to controls	Male AD patients (MMSE 14-22) compared to controls	AD patients (all) compared to controls	AD patients (MMSE > 22) compared to controls	AD patients (MMSE 14-22) compared to controls	Female AD patients compared to controls	Female AD patients (MMSE > 22) compared to controls	Female AD patients (MMSE 14-22) compared to controls	Male AD patients compared to controls	Male AD patients (MMSE > 22) compared to controls	Male AD patients (MMSE 14-22) compared to controls
Number of samples (AD)			79	53	26	44	30	14	35	23	12	79	53	26	44	30	14	35	23	12
Number of samples (Controls)												51	51	51	27	27	27	24	24	24
absolute quantification	Homovanillic acid	Steroid hormones, catecholamines and related	1.13	1.15	1.00	1.22	1.35	1.21	1.03	1.11	0.77	0.228	0.072	0.685	0.115	0.080	0.541	0.881	0.405	0.180
absolute quantification	5-Hydroxy-3-indoleacetic acid (5-HIAA)		1.00	1.09	0.84	1.08	1.21	0.88	0.89	0.95	0.82	0.953	0.317	0.187	0.067	0.013	0.777	0.110	0.467	0.039
absolute quantification	3,4-Dihydroxyphenylacetic acid		1.04	1.08	0.95	1.07	1.08	1.04	1.05	1.07	0.97	0.558	0.293	0.672	0.997	0.638	0.567	0.456	0.345	0.998
absolute quantification	3,4-Dihydroxyphenylglycol (DOPEG)		0.92	0.90	0.96	0.92	0.90	0.95	0.96	0.94	1.02	0.208	0.192	0.529	0.147	0.093	0.627	0.805	0.897	0.713
absolute quantification	Dopamine		1.05	1.04	1.05	1.03	1.03	1.03	1.06	1.05	1.09	0.000	0.000	0.001	0.010	0.015	0.151	0.000	0.008	0.001
absolute quantification	Noradrenaline		1.39	1.34	1.41	1.30	1.28	1.34	1.25	1.24	1.39	0.000	0.001	0.012	0.025	0.035	0.133	0.005	0.011	0.044
absolute quantification	3,4-Dihydroxyphenylalanine (DOPA)		1.12	1.15	1.11	1.13	1.11	1.18	1.16	1.18	1.06	0.022	0.014	0.249	0.047	0.114	0.061	0.224	0.031	0.629
absolute quantification	3-Methoxy-tyrosine		1.04	1.03	1.06	1.01	1.01	1.04	1.08	1.08	1.09	0.255	0.347	0.374	0.306	0.392	0.434	0.579	0.663	0.656
absolute quantification	4-Hydroxy-3-methoxy-phenylglycol (MHPG)		0.83	0.83	0.84	0.81	0.81	0.83	0.86	0.86	0.85	0.003	0.005	0.078	0.041	0.045	0.264	0.039	0.048	0.171
absolute quantification	Histamine		0.87	0.87	0.87	0.87	0.87	0.85	0.82	0.78	0.85	0.008	0.004	0.211	0.235	0.099	0.777	0.005	0.012	0.017
absolute quantification	Testosterone		0.95	0.97	0.93	1.04	1.03	1.07	0.91	0.94	0.88	0.523	0.676	0.423	0.705	0.756	0.713	0.671	0.877	0.431
absolute quantification	21-Hydroxyprogesterone		1.07	1.08	1.05	1.11	1.12	1.04	1.01	1.00	1.02	0.023	0.041	0.064	0.029	0.023	0.205	0.395	0.731	0.104
absolute quantification	Corticosterone		1.12	0.94	1.31	0.84	0.84	0.88	1.31	1.02	1.57	0.619	0.841	0.364	0.886	0.934	0.841	0.323	0.648	0.052
absolute quantification	Cortisol		1.25	1.24	1.37	1.22	1.22	1.26	1.26	1.22	1.39	0.000	0.000	0.000	0.000	0.004	0.003	0.003	0.012	0.009
absolute quantification	Normetanephrine		1.27	1.27	1.27	1.23	1.23	1.19	1.29	1.28	1.32	0.001	0.001	0.032	0.018	0.036	0.086	0.019	0.014	0.215
relative quantification	4-Hydroxyphenylpyruvic acid	Amino acids and related	0.96	0.95	0.97	0.98	1.00	0.97	0.97	0.89	1.01	0.457	0.207	0.689	0.891	0.894	0.594	0.175	0.086	0.967
relative quantification	5-Oxoproline		1.00	1.00	1.00	0.97	0.97	0.96	0.99	0.99	0.98	0.380	0.354	0.665	0.352	0.304	0.676	0.844	0.856	0.894
relative quantification	Alanine		1.07	1.06	1.09	1.12	1.10	1.15	0.96	0.96	1.00	0.176	0.499	0.071	0.130	0.335	0.076	0.634	0.964	0.414
relative quantification	Arginine		1.06	1.03	1.09	1.08	1.02	1.13	1.00	0.99	1.02	0.204	0.444	0.093	0.247	0.549	0.051	0.608	0.638	0.750
relative quantification	Citrulline		1.21	1.21	1.22	1.18	1.16	1.23	1.08	1.09	1.03	0.005	0.015	0.008	0.000	0.003	0.004	0.447	0.530	0.430
relative quantification	Cysteine		1.22	1.22	1.19	1.20	1.25	1.15	1.18	1.18	1.18	0.000	0.000	0.005	0.000	0.000	0.008	0.019	0.017	0.188
relative quantification	Glutamine (incl. Lysine)		1.02	1.02	1.01	1.01	1.03	0.94	1.00	0.95	1.01	0.541	0.665	0.533	0.599	0.766	0.164	0.783	0.418	0.461
relative quantification	Isoleucine		1.01	1.01	1.01	1.12	1.12	1.09	0.95	0.95	0.99	0.224	0.422	0.208	0.196	0.216	0.399	0.573	0.987	0.335
relative quantification	Kynurenine		1.01	1.01	1.00	1.00	1.03	0.99	1.00	0.99	1.05	0.930	0.908	0.989	0.758	0.534	0.809	0.820	0.610	0.718
relative quantification	Leucine		1.10	1.07	1.14	1.18	1.15	1.23	0.99	0.99	1.03	0.099	0.190	0.147	0.105	0.141	0.194	0.393	0.639	0.377
relative quantification	Lysine		0.97	0.97	0.99	0.99	0.99	1.01	0.95	0.95	0.95	0.850	0.979	0.749	0.845	0.881	0.858	0.888	0.944	0.784
relative quantification	Methionine		1.14	1.14	1.09	1.17	1.21	1.10	1.13	1.08	1.15	0.006	0.007	0.076	0.027	0.017	0.311	0.061	0.146	0.079
relative quantification	N,N'-Dimethylarginine		1.06	1.07	1.05	1.13	1.13	1.12	0.99	1.00	0.98	0.128	0.092	0.621	0.067	0.073	0.293	0.830	0.614	0.716
relative quantification	Ornithine (incl. Arginine, Citrulline)		1.10	1.09	1.13	1.21	1.22	1.19	1.02	0.97	1.11	0.013	0.062	0.021	0.002	0.012	0.008	0.751	0.964	0.558
relative quantification	Phenylalanine		1.04	1.03	1.08	1.12	1.11	1.12	1.02	1.01	1.14	0.013	0.044	0.036	0.001	0.002	0.061	0.616	0.758	0.213
relative quantification	Serine		1.11	1.14	1.07	1.12	1.12	1.12	1.11	1.16	1.04	0.006	0.006	0.108	0.054	0.055	0.229	0.057	0.051	0.312
relative quantification	Taurine		0.99	0.98	1.01	1.04	1.05	1.01	0.99	0.94	1.01	0.493	0.578	0.469	0.205	0.131	0.666	0.648	0.360	0.527
relative quantification	Threonine		0.98	1.00	0.98	0.99	0.99	0.98	1.03	1.03	1.02	0.788	0.864	0.770	0.457	0.390	0.820	0.736	0.506	0.836
relative quantification	Tryptophan		1.04	1.02	1.05	1.05	1.07	1.03	1.02	0.99	1.15	0.569	0.712	0.560	0.322	0.094	0.731	0.857	0.222	0.134
relative quantification	Tyrosine		1.08	1.08	1.10	1.13	1.13	1.13	1.03	1.02	1.10	0.033	0.106	0.038	0.023	0.036	0.070	0.403	0.869	0.202
relative quantification	Urea		1.08	1.10	1.01	1.08	1.15	0.93	1.01	0.99	1.04	0.161	0.097	0.509	0.284	0.185	0.656	0.284	0.278	0.550
relative quantification	Valine		0.99	0.99	0.98	1.03	1.05	1.00	0.93	0.93	0.92	0.839	0.953	0.654	0.265	0.310	0.483	0.540	0.325	0.949
relative quantification	1,5-Anhydrosorbitol	Carbohydrates and related	1.04	1.07	1.02	1.12	1.18	0.72	0.95	0.95	0.92	0.940	0.508	0.462	0.503	0.275	0.828	0.392	0.674	0.413
relative quantification	Arabinose		1.11	1.11	1.13	1.12	1.12	1.11	1.13	1.12	1.19	0.021	0.062	0.043	0.035	0.083	0.090	0.233	0.374	0.245
relative quantification	Erythrol		1.08	1.09	1.07	1.10	1.09	1.21	1.05	1.04	1.06	0.013	0.045	0.026	0.048	0.172	0.048	0.137	0.145	0.328
relative quantification	Erythronic acid		1.09	1.09	1.10	1.08	1.05	1.14	1.01	1.00	1.04	0.110	0.107	0.361	0.115	0.192	0.198	0.524	0.368	0.989
relative quantification	Fructose		1.03	1.03	1.07	1.11	1.08	1.17	1.02	1.03	1.02	0.361	0.659	0.213	0.254	0.592	0.118	0.848	0.890	0.855
relative quantification	Galactitol		1.08	1.15	1.07	1.15	1.16	1.06	1.11	1.11	1.13	0.049	0.061	0.170	0.205	0.257	0.379	0.142	0.146	0.307
relative quantification	Glucose		1.01	0.98	1.02	1.01	1.00	1.04	0.99	0.95	1.03	0.657	0.637	0.064	0.334	0.610	0.213	0.709	0.244	0.130
relative quantification	Glucuronic acid		1.01	1.01	1.03	0.98	0.99	0.91	1.00	0.97	1.11	0.736	0.487	0.752	0.530	0.135	0.396	0.867	0.536	0.458
relative quantification	Lyxose		1.11	1.11	1.16	1.15	1.14	1.16	1.15	1.15	1.18	0.013	0.042	0.034	0.028	0.054	0.121	0.186	0.348	0.165
relative quantification	Mannose		1.01	1.00	1.05	1.00	0.99	1.05	0.99	0.99	1.09	0.971	0.867	0.901	0.747	0.641	0.975	0.719	0.764	0.779
relative quantification	myo-Inositol		0.97	0.97	0.97	1.02	0.99	1.09	0.92	0.97	0.90	0.477	0.651	0.443	0.730	0.938	0.379	0.151	0.605	0.036
relative quantification	Ribonic acid		1.08	1.08	1.08	1.18	1.16	1.26	1.02	1.02	1.00	0.049	0.127	0.065	0.020	0.078	0.034	0.712	0.787	0.684
relative quantification	Sorbitol (incl. Mannitol, Galactitol)		1.15	1.13	1.27	1.14	1.14	1.20	1.14	1.09	1.24	0.006	0.094	0.000	0.073	0.238	0.029	0.037	0.255	0.006
relative quantification	scyllo-Inositol		0.79	0.83	0.66	0.84	0.86	0.78	0.60	0.67	0.54	0.016	0.134	0.006	0.730	0.970	0.529	0.001	0.038	0.000
relative quantification	3-Hydroxybutyric acid	Energy metabolism	1.00	1.00	1.02	0.98	0.97	1.03	1.07	1.08	1.05	0.911	0.902	0.635	0.695	0.566	0.918	0.350	0.373	0.537
relative quantification	Citrate		1.03	1.04	1.02	1.02	1.08	1.00	1.00	1.00	1.04	0.486	0.452	0.748	0.339	0.369	0.533	0.812	0.626	0.883
relative quantification	Glycerol-3-Phosphate, polar fraction		1.01	0.93	1.07	0.91	0.88	0.94	1.12	0.99	1.16	0.371	0.220	0.955	0.075	0.092	0.183	0.514	0.953	0.121
relative quantification	Lactate		1.04	1.02	1.05	1.02	1.01	1.04	1.05	1.05	1.06	0.329	0.618	0.234	0.488	0.962	0.260	0.547	0.530	0.722
relative quantification	Pyruvate (incl. Phosphoenolpyruvate)		1.23	1.23	1.19	1.27	1.31	1.05	1.22	1.19	1.27	0.012	0.013	0.078	0.102	0.077	0.336	0.012	0.031	0.028
relative quantification	Carnitine	Miscellaneous	0.95	0.93	0.98	1.22	1.22	1.12	0.82	0.79	0.91	0.632	0.451	0.887	0.310	0.194	0.978	0.110	0.017	0.815
relative quantification	Creatine		1.01	0.99	1.01	1.00	0.95	1.01	1.03	1.03	1.03	0.674	0.820	0.586	0.554	0.736	0.452	0.983	0.973	0.992
relative quantification	Creatinine		1.06	1.05	1.06	1.12	1.14	1.08	1.03	1.00	1.07	0.006	0.029	0.003	0.008	0.022	0.022	0.279	0.611	0.059
relative quantification	Glyceric acid		0.96	0.94	1.04	0.91	0.84	1.00	0.99	1.04	0.95	0.225	0.050	0.635	0.413	0.079	0.253	0.276	0.300	0.430
relative quantification	Hydroxypyridine		1.12	1.14	1.07	1.10	1.17	1.07	1.12	1.11	1.14	0.065	0.055	0.132	0.097	0.071	0.203	0.424	0.576	0.400
relative quantification	Phosphate (inorganic and from organic phosphate		0.98	0.98	0.97	0.91	0.93	0.90	1.04	1.00	1.10	0.569	0.338	0.735	0.989	0.681	0.442	0.268	0.288	0.590
relative quantification	Phosphocreatine		1.00	1.01	0.98	1.06	1.07	1.04	0.97	1.00	0.86	0.628	0.525	0.953	0.503	0.542	0.578	0.891	0.865	0.604
relative quantification	Pseudouridine		1.09	1.09	1.11	1.19	1.18	1.28	0.96	0.97	0.96	0.039	0.083	0.096	0.006	0.028	0.020	0.942	0.937	0.782
relative quantification	putative Tartronic acid		0.97	0.97	0.98	1.05	1.03	1.09	0.86	0.86	0.85	0.186	0.147	0.651	0.770	0.581	0.060	0.025	0.091	0.031
relative quantification	Quinic acid (incl. Chlorogenic acid)		1.33	1.48	1.22	1.22	1.37	1.00	1.90	2.29	1.83	0.002	0.004	0.038	0.114	0.048	0.812	0.005	0.046	0.002
relative quantification	Salicylic acid		1.09	0.93	2.03	1.03	0.90	1.60	1.20	0.99	2.62	0.186	0.854	0.032	0.221	0.485	0.179	0.556	0.542	0.109
relative quantification	Uric acid		1.02	1.06	0.97	1.18	1.22	1.11	0.92	1.02	0.89	0.244	0.150	0.737	0.064	0.028	0.519	0.973	0.990	0.966
relative quantification	Cholesterol	Phosphocholines, sphingomyelins and other lipids	1.03	1.03	1.07	0.97	1.01	0.88	1.17	1.12	1.23	0.220	0.288	0.336	0.327	0.182	0.917	0.404	0.776	0.136
relative quantification	PC C28 + SM C14		0.96	0.97	0.94	0.93	0.93	0.93	1.03	1.04	0.92	0.415	0.195	0.986	0.127	0.097	0.429	0.612	0.836	0.603
relative quantification	PC C30 + SM C16		0.93	0.92	1.01	0.82	0.79	1.00	1.07	1.07	1.03	0.732	0.946	0.410	0.985	0.710	0.490	0.693	0.756	0.709
relative quantification	PC C32 + SM C18		0.99	0.99	1.07	0.92	0.83	1.08	1.26	1.30	1.07	0.952	0.673	0.630	0.692	0.420	0.572	0.796	0.781	0.879
relative quantification	SM C20 + PC C34		1.04	1.03	1.06	1.01	0.99	1.08	1.27	1.30	1.12	0.605	0.846	0.415	0.811	0.829	0.278	0.677	0.616	0.859
relative quantification	SM C22 + PC C36		1.05	1.05	1.10	1.05	1.05	1.11	1.07	1.06	1.08	0.338	0.633	0.169	0.364	0.534	0.255	0.680	0.930	0.480
relative quantification	SM C24 + PC C38		1.04	1.02	1.06	1.02	1.02	1.06	1.08	1.16	1.08	0.378	0.651	0.216	0.443	0.567	0.429	0.664	0.909	0.458
relative quantification	SM C26 + PC C40		1.13	1.11	1.17	1.05	0.97	1.13	1.19	1.23	1.19	0.242	0.415	0.169	0.322	0.491	0.245	0.547	0.633	0.545
relative quantification	Ascorbic acid	Vitamins, cofactors and related	1.06	1.06	1.05	1.09	1.06	1.13	1.09	1.12	1.00	0.505	0.443	0.796	0.409	0.703	0.260	0.928	0.350	0.228
relative quantification	Uridine		0.84	0.81	0.93	0.89	0.83	0.94	0.76	0.74	0.86	0.000	0.000	0.128	0.038	0.014	0.619	0.003	0.002	0.087
relative quantification	Choline		1.03	1.00	1.05	1.25	1.27	1.24	0.91	0.89	0.94	0.179	0.244	0.281	0.003	0.016	0.022	0.099	0.181	0.170
relative quantification	Pantothenic acid		0.97	0.98	0.97	0.98	0.98	0.98	0.95	0.95	0.93	0.619	0.788	0.538	0.656	0.936	0.412	0.813	0.738	1.000
relative quantification	Threonic acid		0.95	0.94	1.01	0.99	0.94	1.10	0.81	0.81	0.82	0.114	0.056	0.853	0.391	0.111	0.116	0.130	0.242	0.096

Legend

abbreviations	
SM	sphingomyelin
PC	phosphocholine

color coding	
	Fold Difference
(pink)	increase by > 33%
(blue)	decrease by > 33%
(grey)	increase/decrease by 10-33%
	p-value
(yellow)	p < 0.01
(light yellow)	0.01 ≤ p < 0.05
(grey)	0.05 ≤ p < 0.1
