# Supplementary material for: Metabolite Profiling of Alzheimer's Disease Cerebrospinal Fluid
Source: PLoS One. 2012 Feb 16;7(2):e31501. doi: 10.1371/journal.pone.0031501 (PMC3281064; doi:10.1371/journal.pone.0031501)
Supplement: Table S2 — The table S2 shows the results as mean of the group values of the metabolites measured in CSF with absolute quantification. All values are in ng/ml. (PDF) [file pone.0031501.s002.pdf]

Supplement Table 2

			Mean of Group Value											
			Female + Male				Female				Male			
			Ctr	AD patients			Ctr	AD patients			Ctr	AD patients		
Quantification method	Metabolite Name	Metabolite Class	Controls (all)	AD patients (all)	AD patients (MMSE > 22)	AD patients (MMSE 14-22)	Female Controls	Female AD patients	Female AD patients (MMSE > 22)	Female AD patients (MMSE 14-22)	Male Controls	Male AD patients	Male AD patients (MMSE > 22)	Male AD patients (MMSE 14-22)
		Number of samples	51	79	53	26	27	44	30	14	24	35	23	12
absolute quantification (ng/ml)	Homovanillic acid	Steroid hormones, catecholamines and related (quantitative values)	72.7	80.9	87.1	69.3	76.7	89.4	94.7	80.0	68.4	71.4	78.9	56.9
absolute quantification (ng/ml)	5-Hydroxy-3-indoleacetic acid (5-HIAA)		53.7	55.5	59.1	48.7	55.6	64.3	68.3	57.2	51.6	45.6	49.2	38.8
absolute quantification (ng/ml)	3,4-Dihydroxyphenylacetic acid		0.91	0.96	1.00	0.88	0.95	0.96	0.99	0.91	0.87	0.96	1.03	0.84
absolute quantification (ng/ml)	3,4-Dihydroxyphenylglycol (DOPEG)		1.30	1.23	1.22	1.25	1.29	1.16	1.13	1.23	1.32	1.31	1.33	1.27
absolute quantification (ng/ml)	Dopamine		0.113	0.120	0.119	0.120	0.114	0.120	0.120	0.119	0.112	0.120	0.118	0.122
absolute quantification (ng/ml)	Noradrenaline		0.22	0.29	0.29	0.29	0.23	0.29	0.29	0.28	0.22	0.3	0.3	0.3
absolute quantification (ng/ml)	3,4-Dihydroxyphenylalanine (DOPA)		0.82	0.92	0.93	0.88	0.77	0.9	0.88	0.92	0.87	0.94	0.99	0.84
absolute quantification (ng/ml)	3-Methoxy-tyrosine		2.87	3.06	3.04	3.11	2.80	3.04	3.01	3.08	2.95	3.09	3.06	3.14
absolute quantification (ng/ml)	4-Hydroxy-3-methoxy-phenylglycol (MHPG)		11.2	9.9	9.8	10.1	11.3	10.1	10.0	10.4	11.1	9.6	9.5	9.7
absolute quantification (ng/ml)	Histamine		0.105	0.093	0.086	0.106	0.110	0.108	0.094	0.133	0.100	0.077	0.077	0.076
absolute quantification (ng/ml)	Testosterone		0.085	0.081	0.082	0.078	0.061	0.060	0.060	0.059	0.111	0.106	0.109	0.101
absolute quantification (ng/ml)	21-Hydroxyprogesterone		0.091	0.097	0.098	0.097	0.088	0.098	0.100	0.094	0.094	0.097	0.096	0.100
absolute quantification (ng/ml)	Corticosterone		0.28	0.30	0.30	0.28	0.27	0.28	0.30	0.24	0.29	0.32	0.30	0.34
absolute quantification (ng/ml)	Cortisol		5.24	6.78	6.64	7.06	5.15	6.79	6.54	7.30	5.33	6.76	6.76	6.76
absolute quantification (ng/ml)	Normetanephrine		0.12	0.15	0.16	0.15	0.12	0.15	0.15	0.15	0.12	0.15	0.16	0.14
